# The Effects of Natural Chinese Medicine Aconite Root, Dried Ginger Rhizome, and* Coptis* on Rectal and Skin Temperatures at Acupuncture Points

**DOI:** 10.1155/2017/7250340

**Published:** 2017-11-12

**Authors:** Jia-Min Yang, Gang Li, Min Wang, Yi-Xi Jin, Feng-Jie Zheng, Yan Sun, Yu-Shan Gao, Shu-Jing Zhang, Peng-Fei Kang, Lin Chen, Meng-Yao Wu, Sheng-Yong Xu, Yu-Hang Li

**Affiliations:** ^1^School of Basic Medical Science, Beijing University of Chinese Medicine, No. 11 Beisanhuan Donglu, Chaoyang District, Beijing 100029, China; ^2^Key Laboratory for the Physics & Chemistry of Nanodevices, Department of Electronics, Peking University, Beijing 100871, China

## Abstract

The 4 properties of Chinese* materia medica* refer to cold, hot, warm, and cool. In the present study, the effects of the* Coptis*, the prepared aconite root, and dried ginger rhizome were compared with regard to the rectal and skin temperature changes of the related body surface acupuncture points (Dazhui, Zhiyang, Mingmen, Zhongwan, and Shenque). The investigation aimed to explore the thermal sensitive points, which can reflect the cold and hot properties of the Chinese herbs. This study showed that the prepared aconite root and dried ginger rhizome exhibited a warming effect on the body temperature, whereas the warming sensitive points were Zhongwan, Shenque, Dazhui, and Zhiyang.* Coptis* exhibited both a warming and a cooling effect on the body temperature, and the cooling sensitive point was Dazhui. The concomitant effect of these three Chinese herbs on the regulation of the body temperature was reflected by Dazhui. However, there are still some limitations and one-sidedness. For instance, the cold and hot property of some herbs cannot be fully reflected through relevant acupoints on the conception and governor vessels. More detecting sites such as ears and internal organs will be selected for further exploration of Chinese herbs' cold and hot property.

## 1. Introduction

Traditional Chinese medicine (TCM) has been used for several thousands of years with documented clinical experience. Due to widespread worldwide use of TCM, certain types of treatment such as Chinese materia medica, acupuncture, moxibustion, massage, and cupping are currently evaluated in the healthcare system as alternative forms of treatment compared with standard medical care. Chinese materia medica is based on the theory of using Chinese medicinal herbs for the therapeutic applications against several forms of disease. Chinese material medica can be classified into 4 categories and 5 types of taste, meridian tropism, ascending and descending, floating and sinking, and poisonous and nonpoisonous properties. The 4 categories of Chinese materia medica refer to cold, hot, warm, and cool properties and can be summarized as cold and hot natural herbal categories. A total of 4 herbal categories are classified according to the reaction of the body following the application of medicinal herbal treatment. The process of treating diseases in TCM is defined as pattern differentiation and treatment* (bian zheng lun zhi)*. The main classification of the syndromes (zheng) is divided into cold and hot syndromes. The drugs that are defined as cold and cool property act by purging heat and lessening the virulence of any pathogenic organism. Thus, they are suitable for diseases of the hot syndromes. Warm and hot drugs dispel cold and invigorate yang, which are mainly indicated for cold syndrome-associated diseases [1].

It has been previously suggested that cold syndrome should be treated by warm and hot medicinal drugs, whereas heat syndromes by cold and cool medicinal drugs. On the basis of this theory, the earliest Pharmaceutical monograph,* Shen Nong's Materia Medica,* formally presented the 4 properties as cold, hot, warm, and cool that referred to the use of Chinese herbs. The 4 properties of the Chinese herbs have been applied in clinical practice for approximately 2,000 years. The application of cold and hot medicinal drugs is contradicted with the nature of the syndrome. According to the description in the Chinese Pharmacopoeia, prepared aconite root* (Radix Aconiti Lateralis Praeparata)* and dried ginger rhizome* (Rhizoma Zingiberis)* belong to the Chinese materia medica with hot properties and are commonly used to treat cold syndromes. In contrast to these herbs,* Coptis (Rhizoma Coptidis)* and common anemarrhena rhizome* (Rhizoma Anemarrhenae)* belong to the Chinese materia medica with cold properties and are commonly used to treat hot syndromes. Tao Hongjing has suggested during the Liang dynasty that the cold and hot properties of the herbs must be clarified, while the flavor of the herbs can be neglected, which emphasized the importance of cold and hot properties on the application of the clinical medication. Therefore, it is important to explore and clarify the cold and/or hot properties of the Chinese materia medica in order to ensure the clinical safety and efficacy of the herbal medicines.

A total of 4 properties have emerged by the prolonged use of TCM in the daily life and long-term clinical practice. The 4 properties of the herbs were summarized according to different reactions to the human body following medication and/or food administration and according to the different therapeutic effects to the diseases. The cold and hot properties of the Chinese herbs are associated with the range of clinical dosage and the nature of each clinical syndrome. The theory that proposes the treatment of the cold syndrome with hot-natured medicine and the treatment of the hot syndrome with cold-natured medicine indicated that the cold and/or hot properties of the medicine were closely related to the cold and/or hot syndromes of the diseases. In the clinic, Chinese herbs that can be used to reduce and/or eliminate hot syndromes are referred to as cold and/or cool-natured herbs, while those that can be used to reduce and/or eliminate cold syndromes are known as hot and/or warm-natured herbs. It has been suggested that “exuberance of yang can induce hot properties, while exuberance of yin can induce cold properties.” The hot syndrome refers to a pathogenic phenomenon of yang exuberance, while the cold syndrome refers to a pathogenic phenomenon of yin exuberance. According to the modern medical practice, the hot syndromes are related to functional hyperactivity, while the cold syndromes are related to functional hypoactivity. Therefore, medicinal drugs that induce cold properties can reduce the pathological hyperactivity, whereas medicinal drugs that induce hot properties can reduce the pathological hypoactivity. The cold and hot properties induced by the clinical application of the Chinese herbs, namely, the treatment of the hot syndrome with hot medicine and the treatment of the cold syndrome with cold medicine, should be taken into consideration in order to avoid the incidence of adverse effects. The medical practitioner Wang Shuhe in the Jin dynasty suggested that patients with yang exuberance syndrome may exhibit adverse effects following administration of Guizhi decoction (a decoction that is used for cold syndrome in* Treatise on Cold Damage (Shang han lun)*), while patients with yin exuberance may not survive following administration of Chengqi decoction (a decoction that is used for the hot syndrome in* Treatise on Cold Damage (Shang han lun)*) [2]. The medical practitioner Xu Dachun in the Qing dynasty suggested that there were differences between prepared aconite root and dried ginger rhizome when used for the same medicinal application with hot properties, while for the same medicinal application of the medicine with cold properties certain differences between the gypsum* (Gypsum Fibrosum)* and* Scutellaria* root* (Radix Scutellariae)* were observed. However the degree of hot and cold properties induced by Chinese herbs was unclear due to the lack of quantitative research methods used for the detection of these properties.

In TCM, the meridian and collaterals exhibit connective and communicative functions between the internal organs and the body surface. The energy of the internal organs can be transmitted to the body surface and/or limbs by the meridian and collaterals. The meridian and collaterals are considered essential for the circulation of the qi and blood, while the acupuncture points are special points used on the surface of the body where the vital energy of the viscera is infused. The functional status of the viscera can be reflected by the corresponding acupuncture points and/or meridians on the surface of the body [3].* Qijing Bamai Kao* has previously stated that the conception vessel is considered to be the sum of yin meridians, which circulate along the median line of the abdomen and have control functions of the body yin meridians. The governor vessel is considered as a pool of yang meridians, which circulate along the posterior median line and regulate the functions of the yang meridians of the body. The acupuncture points Dazhui (GV14), Zhiyang (GV9), Mingmen (GV4), Zhongwan (CV12), and Shenque (CV8) exhibit an association with the qi and the blood components as demonstrated by the therapeutic indications of the meridians, which do not only reflect the state of qi and blood of the body, but also the state of the internal organs. Therefore, in the present study, the hypothesis of specific points in the body that can reflect the cold and/or hot property of Chinese herbs was proposed. The acupuncture points on the conception and governor vessels were selected to explore the biological response that is associated with the cold and hot properties of the Chinese herbs.

Frigid extremities decoction (Si ni tang), which is composed of prepared aconite root, dried ginger rhizome, and liquorice root* (Radix et Rhizoma Glycyrrhizae)* can be used clinically to recuperate depleted yang and rescue the patient from collapse. The compatibilities of the flavor of the herbs (i.e., dried ginger rhizome,* Coptis*,* Scutellaria* root and ginseng* (Radix et Rhizoma Ginseng)*,* Pinellia* tuber* (Rhizoma Pinelliae)*, liquorice root, and Chinese date* (Fructus Jujubae)*) with the hot and/or cold properties can be facilitated by the* Pinellia* Heart-Draining Decoction (ban xia xie xin tang).* Pinellia* tuber and dried ginger rhizome are pungent in flavor and possess hot properties, while* Scutellaria* root and* Coptis* are bitter in flavor and possess cold properties. The compatibility of these 4 herbs can treat epigastric stuffiness and obstruction due to accumulation of cold and hot properties [4]. The dosage and proportion of the medicinal drugs with cold and hot properties can be adjusted by sufficient knowledge of the applications of Chinese herbs by the medical practitioner according to the patient's needs. The precise treatment of each disease requires understanding of the cold and hot properties of the prescription.

Previous studies have attempted to define the degree of cold or hot properties of drugs with regard to the application of Chinese materia medica in TCM clinical treatment [5–8]. A variety of modern scientific technologies have been used to investigate the biomedical effects of the 4 properties, such as animal thermotropism behavior [9, 10], infrared thermal imaging [11], microcalorimetry [12], cytological evaluation [13, 14], biological photon analysis technology evaluation method [15, 16], energy metabolic evaluation method [17, 18], and cold and hot associated protein pathways [19, 20]. However, the quantitative methods used for the evaluation of cold and hot properties of the Chinese materia medica are incomplete and require further investigation.

The 4 properties depend on the reaction of the body following drug administration. The direct cold and/or hot conditions are directly observed by thermoregulation, according to the risen or dropped body temperature. Thermoregulation is considered one of the most important and objective indices used for body temperature changes. We hypothesized that a different thermoregulation response could be expected in animals following administration of Chinese herbs with cold and/or hot properties. Previous studies used infrared thermal camera methodologies in order to detect the body infrared images following dried ginger rhizome (hot-natured herb) and* Scutellaria* root (cold-natured herb) administration and attempted to evaluate the induction of cold and/or hot properties of the Chinese herbs [11]. Yuan et al. [21] established rat models of cold and hot syndromes by separate administration of Chinese herbs with cold and hot properties. The differences between the herbs with cold and hot properties were evaluated by the skin temperature of the nose, eyes, ears, and tails of the rats that were detected by infrared thermal images. Hong et al. [22] used a thermometer in order to record the rectal temperature of mice following Panax Ginseng and* Panax quinquefolius* administration under different environmental temperature conditions. Their study aimed to evaluate the effects of different environmental temperature conditions and herbal medicine administration on the body temperature.

Bach et al. [23] compared the differences noted between conductive and infrared devices that were used for the measurement of the mean skin temperature during the resting state, the exercise in the presence heat, and the recovery state. The authors concluded that infrared thermal imaging was not suitable for the detection of the motion status, while thermistor use resulted in high accuracy during resting and motion states. The use of the thermometer was not convenient for real-time monitoring and recording. Consequently, this present study utilized the thermistor equipment that has the advantage of detecting the body temperature for 24 hours following medicinal drug administration. Compared with the previous experimental method, this second method moreover excludes animal anesthesia interference.

In the current study, the characteristics of the cold and hot properties of the Chinese herbs were examined using the concept of the traditional Chinese medicine, and the acupuncture point specific theory. The study aimed to compare the different effects of prepared aconite root, dried ginger rhizome, and* Coptis* on the body temperature changes and to explore the sensitive points of the body that can reflect the herbal cold and hot properties. Rectal and skin temperature changes of Dazhui (GV14), Zhiyang (GV9), Mingmen (GV4), Zhongwan (CV12), and Shenque (CV8) acupuncture points were recorded for 24 hours prior to and following medicinal herb administration by the thermistor dynamic device.

## 2. Materials and Methods

### 2.1. Chinese Medicinal Herbs

Prepared aconite root, dried ginger rhizome, and* Coptis* were purchased from Beijing Tong Ren Tang Pharmacy (Beijing, China).

### 2.2. Animals and Grouping

A total of 33 adult male rabbits weighing 2.5 kg ± 0.5 kg were used in the present study. They were housed in a room that was maintained at a constant temperature of 23 ± 1°C, a constant humidity of 45p.100 ± 5p.100, and a 12 h : 12 h light/dark cycle with light onset at 08:00 am. Drinking water and laboratory rodent chow were provided ad libitum. All studies on unanesthetized rabbits were conducted in the facilities of Beijing University of Chinese Medicine and were approved by the Institutional Animal Care and Use Committee (BUCM-3-2015032502-1002).

A total of 33 rabbits were randomly divided into 6 groups, including prepared aconite root (PAR) group (dosage A group (*n* = 4), dosage B group (*n* = 5)), dried ginger rhizome (DGR) group (dosage A group (*n* = 6), dosage B group (*n* = 6)), and* Coptis* (C) group (dosage A group (*n* = 6), dosage B group (*n* = 6)).

### 2.3. Preparation of Decoction

Initially the ingredients were placed in an earthenware pot and clean water was added until all the ingredients were covered. The ingredients were soaked for 60 min. Subsequently, the solution was boiled and simmered and the decoction was conducted by pouring water to the remaining precipitates. The new solution was heated repeatedly as described previously. The two decoctions were mixed together and the final concentrations of the herbal medicinal extracts were 1 g·ml^−1^ and 2 g·ml^−1^.

### 2.4. Herbal Dosage and Administration

The clinical herbal dosage conversion between human and rabbit was estimated based on a previous study [24]: human herbal dosage (g·kg^−1^): rabbit herbal dosage (g·kg^−1^) = 3.08 : 1. Provided that a person (60 kg) receives 10 g of each herb, the equivalent dose for a rabbit will be 0.513 g·kg^−1^. However the dosage for the rabbits used in the present study was 10-fold higher (dosage A, 5.13 g·kg^−1^) and 20-fold higher (dosage B, 10.26 g·kg^−1^).

The herbs were administered intragastrically to the rabbits.

### 2.5. Apparatus

*Pt100 Thermistor*. The temperature range used was between −50°C and 200°C. The accuracy was ± 0.2°C. The appearance parameters of the chip type thermistor and cylindrical thermistor were 10 mm × 10 mm × 2 m and 3 mm × 19 mm × 2 m (diameter × length × line length) separately. The apparatuses were purchased from Shanghai Xinghua Xikai Equipment Co., Ltd (Shanghai, China).

*Paperless Recorder*. The screen used was 7 inch true color TFTLCD (800 × 480 dot matrix) and the accuracy was ±0.2p.100 F.S. The recorder was purchased from Shanghai Xinghua Xikai Equipment Co., Ltd (Shanghai, China).

The temperature and humidity were set at a constant level of 26°C ± 1°C and 55p.100 ± 5p.100.

### 2.6. Acupuncture Points Location

The acupuncture points were located as follows.

*Dazhui (GV14)*. The point was located in the posterior region of the neck, in the depression inferior to the spinous process of the seventh cervical vertebra (C7), on the posterior median line.

*Zhiyang (GV9)*. The point was located in the upper back region, in the depression inferior to the spinous process of the seventh thoracic vertebra (T7), on the posterior median line.

*Mingmen (GV4)*. The point was located in the lumbar region, in the depression inferior to the spinous process of the second lumbar vertebra (L2), on the posterior median line.

*Zhongwan (CV12)*. The point was located on the upper abdomen, on the line between the xiphisternal junction and pubic symphysis (a total of 13 equal parts), 4 equal parts inferior to the xiphisternal junction.

*Shenque (CV8)*. The point was located on the upper abdomen, on the line between the xiphisternal junction and pubic symphysis (a total of 13 equal parts), 5 equal parts superior to the pubic symphysis. The location of the acupuncture points was based on previous studies [25].

### 2.7. Procedure

**Figure 1:**
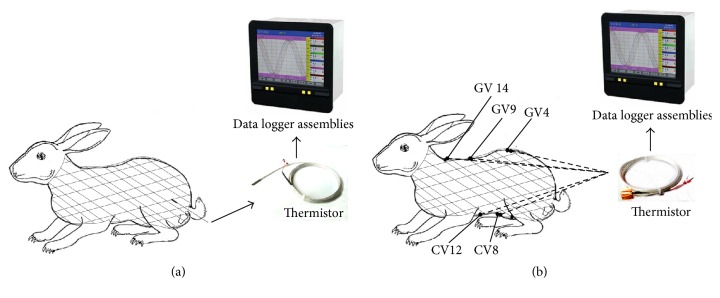
The procedure of temperature detection. (a) Rectal temperature detection. (b) Skin temperature detection.

The front and back fur of the rabbits was shaved by an electric hair clipper 24 hours prior to temperature detection. On the detection day, the acupuncture points were marked with a marker. Chip shaped thermistors were attached to the Dazhui (GV14), Zhiyang (GV9), Mingmen (GV4), Zhongwan (CV12), and Shenque (CV8) acupuncture points. A cylindrical thermistor was inserted into the rectum for 150 mm. The perirectal area was covered with gauze in order to prevent loss of contact with the thermistor. The rabbits were wrapped with medical hollow elastic bandage. Following administration of water, the rabbits were put into a cage. The body temperature of the rabbits was recorded for 24 h at a constant temperature and humidity environment (with temperature 26°C ± 1°C, humidity 60p.100 ± 5p.100). On the third day following medicinal herb administration, the skin temperature of the acupuncture points and the rectal temperature were recorded for 24 h. See Figure 1.

### 2.8. Statistical Analysis

Microsoft Excel 2010 software was used to calculate the temperature difference of the acupuncture points and rectum between medicinal herb administration and water administration. The temperature difference was defined as follows: Δ*T*, Δ*T* = *T*_herbs_ − *T*_water_. A temperature difference of higher than 0°C (Δ*T* > 0°C) indicated that body temperature following herbal administration was superior to that following water administration, while a temperature difference of lower than 0°C (Δ*T* < 0°C) indicated that the body temperature following herbal administration was inferior to that following water administration. The mean temperature changes of the detected sites in each group were analyzed.

## 3. Results

### 3.1. The Effects of PAR, DRG, and C Dosage Groups on the Rectal Temperature Changes

The rectal temperature rose in both PAR A and PAR B dosage groups within 24 h of herbal administration (with Δ*T* > 0°C). The maximum rise in the temperature of the PAR A dosage group was 0.65°C following 13 h of herbal administration, while the maximum rise in the temperature of PAR B dosage group was 0.54°C following 11 h of herbal administration.

The rectal temperature rose in both DGR A and DGR B dosage groups within 24 h of herbal administration (with Δ*T* > 0°C). The maximum temperature of the DGR A dosage group was 0.9°C following 3.1 h of herbal administration, while the maximum temperature of the DGR B dosage group was 0.75°C following 16 h of herbal administration.

**Figure 2:**
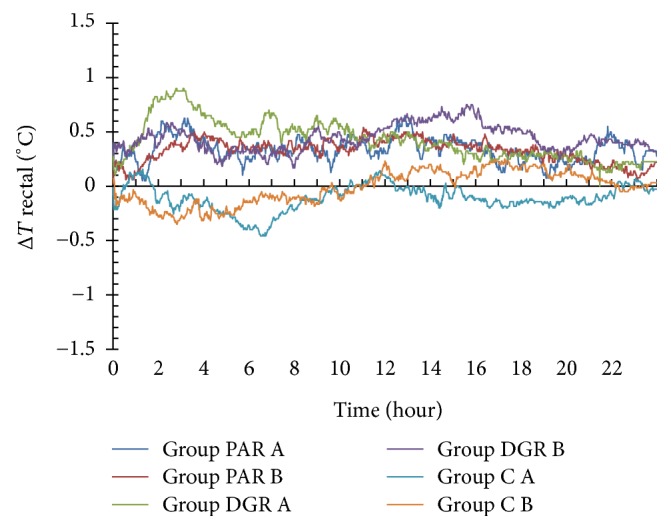
Rectal temperature changes within 24 h following herbal administration of PAR, DRG, and C (A and B) dosage groups.

The rectal temperature dropped in the C A dosage group within 24 h following herbal administration (with Δ*T* < 0°C). The minimum temperature was −0.46°C following 6.75 h of herbal administration. The rectal temperature dropped in the C B dosage group at 11.05 h following herbal administration (with Δ*T* < 0°C), whereas it was risen during the 11.1 h–22.17 h time interval (with Δ*T* > 0°C). The minimum temperature noted was −0.35°C at 2.83 h following herbal administration. See Figure 2.

### 3.2. The Effects of PAR, DRG, and C Dosage Groups on the Skin Temperature Changes of the Acupuncture Point Dazhui (GV14)

The skin temperature of the acupuncture point Dazhui (GV14) in the PAR A and PAR B dosage groups rose within 24 h following herbal administration (with Δ*T* > 0°C), whereas the maximum temperature changes were 1.7°C at 12.67 h following herbal administration. The skin temperature of the Dazhui (GV14) acupuncture point in the PAR B dosage group dropped within 3.3 h following herbal administration (with Δ*T* < 0°C) and rose during the 3.3 h–24 h time interval (with Δ*T* > 0°C). The maximum temperature change was 0.88°C at the 13.62 h time period following herbal administration.

The skin temperature of the Dazhui (GV14) acupuncture point in DGR A and B dosage groups DGR rose within 24 h following herbal administration (with Δ*T* > 0°C), whereas the maximum temperature changes were 0.95°C at the time point of 3.22 h and 1.22°C at the time point of 15.75 h, following herbal administration.

**Figure 3:**
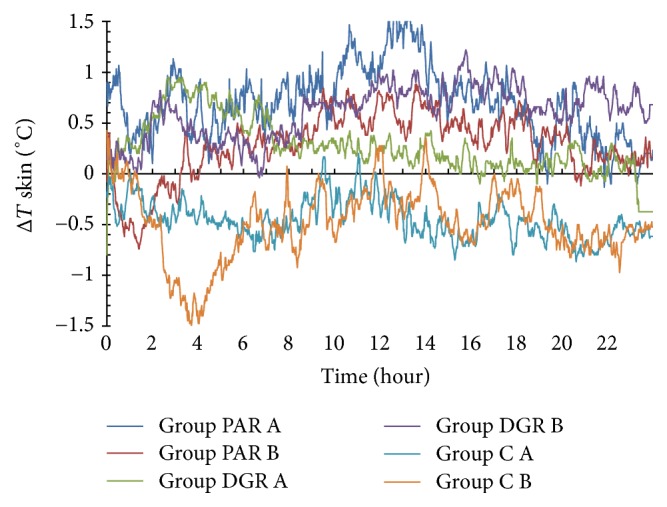
Skin temperature changes of the Dazhui (GV14) acupuncture point within 24 h of herbal administration of PAR, DRG, and C (A and B) dosage groups.

The skin temperature of the acupuncture point Dazhui (GV14) in C A and B dosage groups dropped within 24 h of herbal administration (with Δ*T* < 0°C), whereas the minimum temperature changes were −0.77°C at 6.5 h and −1.5°C at 3.73 h, following herbal administration. See Figure 3.

### 3.3. The Effects of PAR, DRG, and C Dosage Groups on the Skin Temperature Changes of the Acupuncture Point Zhiyang (GV9)

The skin temperature changes of the Zhiyang (GV9) acupuncture point in the PAR A dosage group rose during the 9 h–24 h interval periods (with Δ*T* > 0°C), whereas the maximum value was 1.15°C at 12.72 h following herbal administration. The skin temperature changes of the Zhiyang (GV9) acupuncture point in the PAR B dosage group dropped within 3.3 h following herbal administration (with Δ*T* < 0°C) and rose at the 3.3 h–24 h time interval (with Δ*T* > 0°C). The maximum value was 0.72°C at 13.58 h following herbal administration.

The skin temperature of the acupuncture point Zhiyang (GV9) in the DGR A and B dosage groups rose within 24 h following herbal administration (with Δ*T* > 0°C) and the maximum temperature changes were 1.28°C at 3.32 h and 1.1°C at 15.58 h following herbal administration.

**Figure 4:**
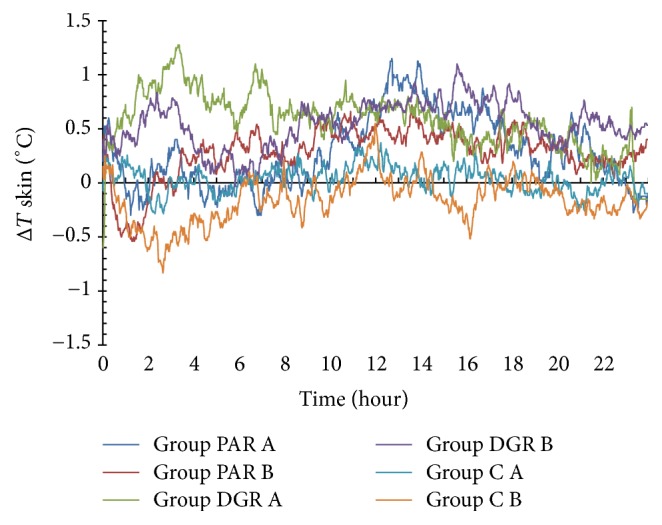
Skin temperature changes of the Zhiyang (GV9) acupuncture point within 24 h following herbal administration of the PAR, DRG, and C (A and B) dosage groups.

The skin temperature changes of the acupuncture point Zhiyang (GV9) in the C A dosage group remained at 0°C with minor fluctuations and the minimum temperature value was −0.29°C at 2.67 h following herbal administration. The skin temperature changes of the acupuncture point Zhiyang (GV9) in the C B dosage group dropped within 24 h following herbal administration with the exception of the 11.15 h–12.73 h time interval (with Δ*T* < 0°C). The minimum temperature change was −0.83°C at 2.67 h following herbal administration. See Figure 4.

### 3.4. The Effects of PAR, DRG, and C Dosage Groups on the Skin Temperature Changes of the Acupuncture Point Mingmen (GV4)

The skin temperature changes of Mingmen (GV4) in the PAR A dosage group rose at the 9 h–24 h time intervals (with Δ*T* > 0°C) and the maximum temperature value was 1.03°C at 13.83 h following herbal administration. The skin temperature changes of Mingmen (GV4) in the PAR B dosage group dropped within 3.3 h following herbal administration (with Δ*T* < 0°C) and rose at the 3.3 h–24 h time interval (with Δ*T* > 0°C). The maximum temperature value was 0.46°C at the 13.67 h following herbal administration.

The skin temperature changes of the Mingmen (GV4) acupuncture point in the DGR A dosage group rose within 14.27 h following herbal administration (with Δ*T* > 0°C) and dropped at the 14.28 h–24 h time interval (with Δ*T* < 0°C). The maximum temperature value was 0.9°C at 6.67 h following herbal administration. The skin temperature changes of the Mingmen (GV4) acupuncture point in the DGR B dosage group rose within 24 h following herbal administration (with Δ*T* > 0°C). The maximum value was 0.75°C at 15.67 h following herbal administration.

**Figure 5:**
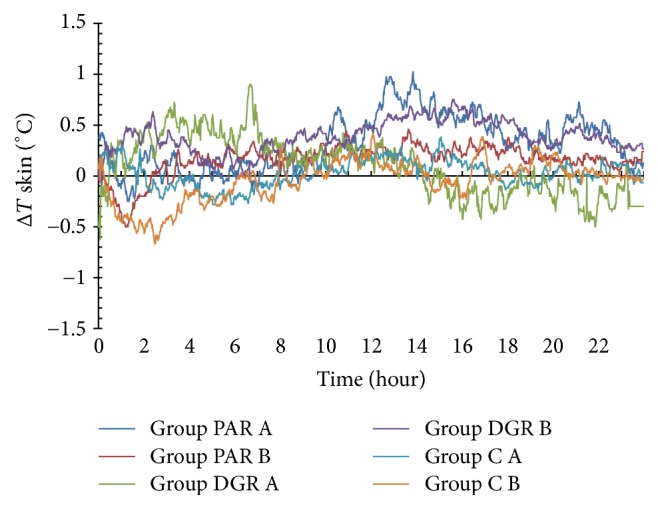
Skin temperature changes of the Mingmen (GV4) acupuncture point within 24 h of herbal administration of the PAR, DRG, and C (A and B) dosage groups.

The skin temperature of the Mingmen (GV4) acupuncture point in the C A dosage group dropped at the 2.08 h–9.45 h time interval (with Δ*T* < 0°C) and rose at the 9.47 h–17.27 h time interval (with Δ*T* > 0°C) following herbal administration. The minimum value was −0.28°C at 5.83 h following herbal administration. The skin temperature of the Mingmen (GV4) acupuncture point in the C B dosage group dropped within 9.08 h (with Δ*T* < 0°C), and remained at approximately 0°C during the 9.1 h–24 h time interval. The minimum temperature value was −0.67°C at 2.5 h following herbal administration. See Figure 5.

### 3.5. The Effects of PAR, DRG, and C Dosage Groups on the Skin Temperature Changes of the Acupuncture Point Zhongwan (CV12)

The skin temperature changes of the Zhongwan (CV12) acupuncture point in PAR A and B dosage groups rose within 24 h following herbal administration (with Δ*T* > 0°C). The maximum temperature values of the PAR A and B groups were 1.73°C at 14.25 h and 0.98°C at 9 h, following herbal administration.

The skin temperature changes of the Zhongwan (CV12) acupuncture point in the DGR A and B dosage groups rose within 24 h following herbal administration (with Δ*T* > 0°C). The maximum temperature values were 1.23°C at 4.75 h and 0.98°C at 15.83 h, following herbal administration.

**Figure 6:**
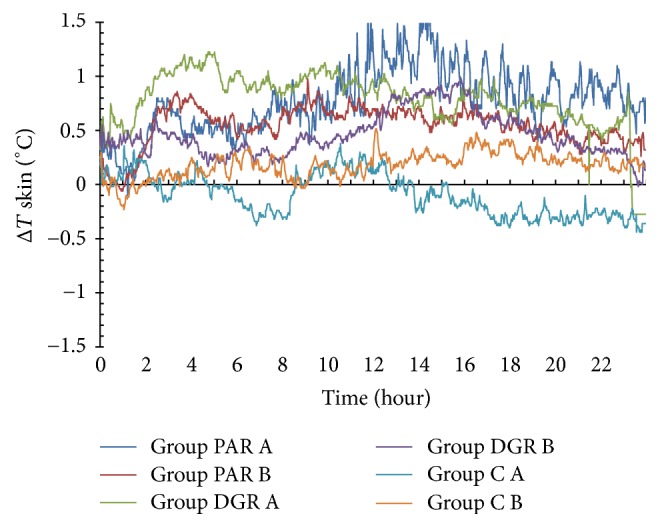
Skin temperature changes of the Zhongwan (CV12) acupuncture point within 24 h of herbal administration of the PAR, DRG, and C (A and B) dosage groups.

The skin temperature change of the Zhongwan (CV12) acupuncture point in the C A dosage group was approximately 0°C within 4.65 h following herbal administration and it dropped during the 4.67 h–8.57 h interval and during the 12.75 h–24 h interval (with Δ*T* < 0°C). The aforementioned temperature change rose at the 9 h–12.73 h time interval (with Δ*T* > 0°C), whereas the minimum temperature value was −0.36°C at 7 h following herbal administration. The skin temperature changes of the Zhongwan (CV12) acupuncture point in the C B dosage group dropped during the 0.72 h–1.83 h interval (Δ*T* < 0°C) and rose during the 1.85 h–24 h interval (with Δ*T* > 0°C). See Figure 6.

### 3.6. The Effects of the PAR, DRG, and C Dosage Groups on the Skin Temperature Changes of the Acupuncture Point Shenque (CV8)

The skin temperature of the Shenque (CV8) acupuncture point in the PAR A dosage group dropped within 1.95 h (with Δ*T* < 0°C) and rose during the 1.95 h–24 h interval (with Δ*T* > 0°C) following herbal administration. The maximum temperature value was 1.5°C at 14.25 h. The skin temperature of the Shenque (CV8) acupuncture point in the PAR B dosage group dropped during the 0.32 h–1.83 h interval (with Δ*T* < 0°C) and rose during the 1.83 h–24 h interval (with Δ*T* < 0°C) following herbal administration. The maximum temperature value was 1.12°C at 9 h following herbal administration.

The skin temperature changes of the Shenque (CV8) acupuncture point in the DGR A and B dosage groups rose within 24 h of herbal administration (with Δ*T* > 0°C) and the maximum temperature values were 0.98°C at 10.37 h and 1.17°C at 14 h.

**Figure 7:**
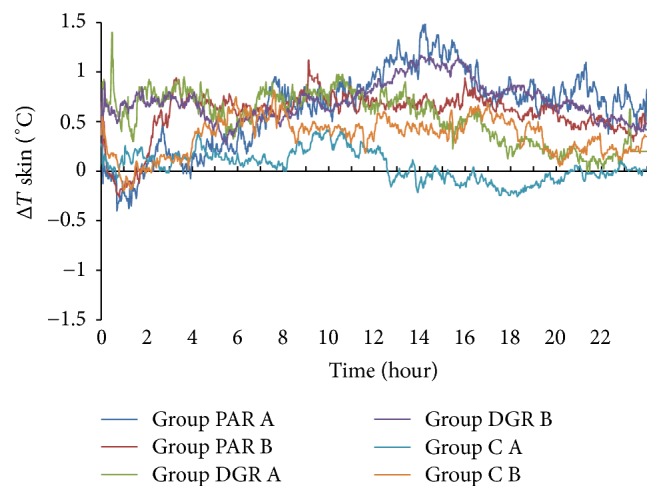
Skin temperature changes of the Shenque (CV8) acupuncture point within 24 h of herbal administration of the PAR, DRG, and C (A and B) dosage groups.

The skin temperature changes of the Shenque (CV8) acupuncture point in the C A dosage group rose within 12.6 h of herbal administration (with Δ*T* > 0°C) and dropped at the 12.6 h–24 h time interval (with Δ*T* < 0°C). The minimum temperature value was −0.26°C at 18.33 h following herbal administration. The skin temperature changes of the Shenque (CV8) acupuncture point in the C B dosage group dropped during the 0.72 h–1.83 h time interval (with Δ*T* < 0°C) and rose during the 1.85 h–24 h interval (with Δ*T* > 0°C). The minimum value was −0.2°C following 1 h of herbal administration. See Figure 7.

## 4. Discussions

In the present study, the different effects of prepared aconite root, dried ginger rhizome, and* Coptis* were compared with regard to the body temperature changes, whereas the sensitive points of the body that can reflect their cold and hot properties were further explored. The rectal and skin temperature of the Dazhui (GV14), Zhiyang (GV9), Mingmen (GV4), Zhongwan (CV12), and Shenque (CV8) acupuncture points were detected by a thermistor dynamic device at 24 h prior to and following medicinal herb administration.

### 4.1. The Effects of Prepared Aconite Root (PAR) on the Rectal and Skin Temperature Changes of the Acupuncture Points

The results indicated that the rectal and skin temperature of the Dazhui (GV14), Zhiyang (GV9), Mingmen (GV4), Zhongwan (CV12), and Shenque (CV8) acupuncture points rose following herbal administration.

The thermal sensitive points on the surface of the body in the PAR A and B dosage groups manifested as Zhongwan (CV12) and Shenque (CV8) separately.

The heating intensities of each detection site in the PAR A dosage group were decreased in turn as follows: Zhongwan (CV12, Δ*T*_max_ = 1.73°C), Dazhui (GV14) (Δ*T*_max_ = 1.7°C), Shenque (CV8) (Δ*T*_max_ = 1.5°C), Zhiyang (GV9) (Δ*T*_max_ = 1.15°C), and Mingmen (GV4) (Δ*T*_max_ = 1.03°C). The rectal temperature change was Δ*T*_max_ = 0.65°C. The heating intensities of each detection site in the PAR B dosage group were decreased in turn as follows: Shenque (CV8, Δ*T*_max_ = 1.12°C), Zhongwan (CV12, Δ*T*_max_ = 0.98°C), Dazhui (GV14, Δ*T*_max_ = 0.88°C), Zhiyang (GV9, Δ*T*_max_ = 0.72°C), and Mingmen (GV4, Δ*T*_max_ = 0.46°C). The rectal temperature change was Δ*T*_max_ = 0.54°C.

### 4.2. The Effects of Dried Ginger Rhizome (DGR) on the Rectal and Skin Temperature Changes of the Acupuncture Points

The results indicated that the rectal and skin temperature of the Dazhui (GV14), Zhiyang (GV9), Mingmen (GV4), Zhongwan (CV12), and Shenque (CV8) acupuncture points rose following herbal administration. The thermal sensitive points on the surface of the body in the DGR A and B dosage groups manifested as the Zhiyang (GV9) and Dazhui (GV14) acupuncture points.

The heating intensities of each detection site in the DGR A dosage group were decreased in turn as follows: Zhiyang (GV9, Δ*T*_max_ = 1.28°C), Zhongwan (CV12, Δ*T*_max_ = 1.23°C), Shenque (CV8, Δ*T*_max_ = 0.98°C), Dazhui (GV14, Δ*T*_max_ = 0.95°C), and Mingmen (GV4, Δ*T*_max_ = 0.9°C). The rectal temperature change was Δ*T*_max_ = 0.9°C. The heating intensities of each detection site in the FGR B dosage group were decreased in turn as follows: Dazhui (GV14, Δ*T*_max_ = 1.22°C), Shenque (CV8, Δ*T*_max_ = 1.17°C), Zhiyang (GV9, Δ*T*_max_ = 1.1°C), Zhongwan (CV12, Δ*T*_max_ = 0.98°C), and Mingmen (GV4, Δ*T*_max_ = 0.75°C). The rectal temperature change was Δ*T*_max_ = 0.75°C.

### 4.3. The Effect of* Coptis* (C) on the Rectal and Skin Temperature Changes of the Acupuncture Points

The results indicated that the rectal and skin temperature of the Dazhui (GV14) and Zhongwan (CV12) acupuncture points dropped following herbal administration, while the skin temperature changes of the Mingmen (GV4) acupuncture point manifested by a drop and a subsequent rise. The skin temperature changes of the Shenque (CV8) acupuncture point manifested by an initial rise and a subsequent drop. No prominent temperature changes were noted with regard to the Zhiyang (GV9) acupuncture point. The rectal and skin temperature changes of the Mingmen (GV4) acupuncture point in the C B dosage group were detected by an initial drop and a subsequent rise, whereas the skin temperature changes of the Dazhui (GV14) and Zhiyang (GV9) acupuncture points were detected by a drop and the skin temperature changes of the Zhongwan (CV12) and Shenque (CV8) acupuncture points by a rise. The temperature of the acupuncture points varied according to the dose of the medicinal herb. The thermal effects in the C A and B dosage groups exhibited concomitant rise and drop in the temperature. The thermal sensitive point was defined as the Dazhui (GV14) acupuncture point in group C.

The cooling intensities of each detection site in the C A dosage group were decreased in turn as follows: Dazhui (GV14, Δ*T*_min_ = − 0.77°C), rectal temperature (Δ*T*_min_ = −0.46°C), Zhongwan (CV12, Δ*T*_min_ = −0.36°C), Zhiyang (GV9, Δ*T*_min_ = −0.29°C), Mingmen (GV4, Δ*T*_min_ = −0.28°C), and Shenque (CV8, Δ*T*_min_ = −0.26°C).

The cooling intensities of each detection site in the C B dosage group were decreased in turn as follows: Dazhui (GV14, Δ*T*_min_ = −1.5°C), Zhiyang (GV9, Δ*T*_min_ = −0.83°C), Mingmen (GV4, Δ*T*_min_ = −0.67°C), rectal temperature (Δ*T*_min_ = −0.35°C), Zhongwan (CV12, Δ*T*_min_ = −0.23°C), and Shenque (CV8, Δ*T*_min_ = −0.2°C).

In summary, the effects of prepared aconite root and dried ginger rhizome on the temperature changes notably manifested as a warming up effect, and the thermal sensitive points on the body surface were the Zhongwan (CV12), Shenque (CV8), Dazhui (GV14), and Zhiyang (GV9) acupuncture points. The effect of* Coptis* on the temperature changes exhibits a simultaneous rise and drop, and the thermal sensitive point on the body surface was the Dazhui (GV14) acupuncture point. The present study indicated that the effects of Chinese herbs with cold and/or hot properties on the body temperature changes were noted on the Dazhui (GV14) acupuncture point.

In Chinese medicine, the use of prepared aconite root affects the meridians of the kidney, heart, and spleen organs and acts by recuperating the depleted yang and rescuing the patient from collapse. This effect supplements the “fire of the gate of life” and restores yang, expelling cold in order to relieve pain. Dried ginger rhizome affects the meridians of spleen, stomach, heart, and lung organs, which exert the functions of warming at the middle energizer meridian and dispelling cold. This results in recuperating the depleted yang and warming the lungs to resolve fluid retention. The* Coptis* herb affects the meridians of heart, liver, stomach, and large intestine organs and exerts functions of heat clearance and dampness elimination. In addition, it eliminates pathogenic fire and aids the detoxication [26]. In the traditional meridian and collateral theories, acupoints Zhongwan (CV12) and Shenque (CV8) are located in the conception vessel. Zhongwan (CV12) is the Front-Mu point of the stomach and the influent point of all the fu organs, which acts by strengthening the spleen and the stomach and promoting digestive function, thus replenishing the Qi of the Middle energizer and tranquilizing the mind [27]. Recent studies indicated that Zhongwan (CV12) acupuncture point is associated with related acupuncture points in the conception vessel, governor vessel, and bladder meridian of the foot taiyang, such as acupoints Feishu (BL13), Xinshu (BL15), Ganshu (BL18), Pishu (BL20), Shenshu (BL23), and Dachangshu (BL25) [28]. Therefore, Zhongwan (CV12) is very effective for the treatment of the disorders associated with these related* zang fu* organs. Acupoint Shenque (CV8) exhibits the function of warming the middle energizer meridian in order to relieve diarrhea, recuperating yang, avoiding yang exhaustion and identifying specific channels in order to stop pain. It is closely associated with the 12 meridians and internal organs. The Shenque (CV8) acupuncture point is the stimulus for the regulation of the dysfunction of* zang fu* organs and meridians in addition to its function as a reaction point of physiological and pathological changes of viscera, meridian and collaterals, qi, and blood [29]. The Zhiyang (GV9) and Dazhui (GV14) acupuncture points are located on the governor vessel and are considered the yang vigorous points. The Zhiyang (GV9) acupuncture point has the function of exciting yang qi. It can reinforce qi and strengthen yang, nourish the blood, and quicken the collaterals by moxibustion. The functions of the latter aforementioned acupuncture point are notably the excitation of yang qi of the whole body, the warming and dissolution of the cold properties, the release of the channels, the quickening of the collaterals, and the redirection of the qi flow in order to loosen the center that is stimulated by acupuncture [30]. The Dazhui (GV14) acupuncture point is considered the connecting point of the governor vessel and the three yang meridians of the hand and foot, which are essential in the excitation of the yang qi. It is the commonly used heat clearing and yang qi reinforcing point in the clinic [31]. In the present study, the thermal sensitive points were reflected on the body surface by prepared aconite root, dried ginger rhizome, and* Coptis* in order to adjust their application to the description of the meridian tropism theory of the Chinese medicine. It states that the skin temperature changes prominently appear at the Zhongwan (CV12), Shenque (CV8), Dazhui (GV14), and Zhiyang (GV9) acupuncture points. It has been shown that the regulation of the internal organs by the drugs is closely related to the meridian tropism and clinical efficacy. The present study further confirms that the theory of traditional Chinese medicine is related to the theory of acupuncture points and internal organs, which provides evidence for the scientific use of this type of medicine in the clinic.

In TCM, meridian and collaterals have connective and communicative functions between internal organs and the body surface. The meridian and collaterals are considered the key components required for the circulation of the qi and blood, while the acupuncture points are notably the points on the surface of the body where the vital energy of the viscera is infused. The functional status of the viscera can be reflected by the corresponding acupuncture points and/or meridians on the surface of the body [3]. In the present study, the cold/hot properties and meridian tropism of the Chinese herbs were reflected by the corresponding acupuncture points. This confirmed an association between the meridian tropism of medicine and the acupuncture points that was in agreement with the hypothesis of the acupuncture points/meridians and* zang fu* organs.

A similar study focused on the application of an infrared thermal camera in order to detect the skin temperature of the lower abdomen, uterus area, governor vessel, and Shenque (CV8) acupuncture point so as to evaluate the drug targeting of the Left-Restoring Pill (zuo gui wan) and of the Right-Restoring Pill (you gui wan). The results indicated that the drug targeting was focused on the governor vessel and the Shenque (CV8) acupuncture point, which demonstrated that the cold and hot properties caused by the drug treatment exerted a clear reaction site on the body [32]. The results of the aforementioned study were consistent with the results of the present study that further explored the specific response of a single herbal medicine on the governor and conception vessels and provided an experimental basis for the effects of different cold and hot herb-induced medical properties caused to the body.

One way to regulate the body temperature is through blood supply towards the skin. When the body temperature decreased, the blood vessel will constrict and a mass of heat will be taken from the skin, leading to the decreased skin temperature. In the same way, when the body temperature increased, blood will flow to the skin surface, leading to a rise of skin temperature. In a stable environment, the average body temperature can predict the body's thermal response [33]. In this study, the effects of prepared aconite root and dried ginger rhizome on the rectal and skin temperature changes were mainly manifested as warming effects, while the effects of* Coptis* on the rectal and skin temperature changes were mainly manifested as cooling effects. This may be related to the hot property of prepared aconite root and dried ginger rhizome, which can dilate blood vessels and lead to rise of the skin temperature. In contrast, the cold property of* Coptis* may induce vasoconstriction and skin temperature drop.

## 5. Conclusion

In this study, thermistor dynamic device was applied to explore the effect of cold/hot-natured Chinese herbs on the skin surface of corresponding acupoints, which preliminarily proved the objective existence of specific points reflecting the cold/hot property of Chinese herbs, and the dynamic thermoregulation changes were partly observed after herbal administration. The thermoregulation of cold/hot-natured Chinese herbs can be reflected on different acupoints. Furthermore, cold/hot-natured Chinese herbs have their own specific sensitive surface points. Both cold and hot-natured Chinese medicine can cause changes of the body temperature including rise and drop.

This study accumulated rich experience in the methodology exploration of cold and hot property in TCM. However, there are still some limitations and one-sidedness. For instance, the cold and hot property of some herbs cannot be fully reflected through relevant acupoints on the conception and governor vessels, which illustrated the complexity of Chinese herbal properties and their multisystem regulation on the body. This study provides experimental evidence for methodology exploration of Chinese herbs' four properties, and more sites will be chosen in our further study, including acupoints, ears, and internal organs.
